# The risk of upper gastrointestinal bleeding associated with concomitant proton pump inhibitor administration during dual antiplatelet therapy with aspirin and prasugrel: a retrospective single-center study

**DOI:** 10.1186/s40780-024-00398-y

**Published:** 2024-11-25

**Authors:** Yutaro Ide, Go Morikawa, Kyohei Yoshida, Yuki Takano, Ken Kubota, Katsuko Okazawa, Takeo Yasu

**Affiliations:** 1 Department of Pharmacy, Hokushin General Hospital, 1-5-63, Nishi, Nakano, Nagano, 383-8505 Japan; 2 Department of Medicinal Therapy Research, Pharmaceutical Education and Research Center, Meiji Pharmaceutical University, Tokyo, Japan; 3 Present address: Division of Legal Medicine, Department of Forensic Science, Iwate Medical University, 1-1-1 Idaidori, Yahaba-cho, Shiwa-gun, Iwate 028-3694 Japan

**Keywords:** PCI, DAPT, PPI, UGBI, Prasugrel, Lansoprazole, Esomeprazole

## Abstract

**Objective:**

Dual‐antiplatelet therapy (DAPT) and proton pump inhibitor (PPI) are frequently prescribed after percutaneous coronary intervention (PCI) with drug-eluting stents (DES) placement. However, studies that evaluate the optimal PPI when used as primary prevention in patients without a history of peptic ulcer disease or upper gastrointestinal bleeding (UGIB), particularly in the context of DAPT involving prasugrel, are lacking. This study aimed to assess the efficacy and safety of PPI use in preventing UGIB in this patient population.

**Methods:**

This study included patients who underwent PCI with coronary stent placement for acute coronary syndrome or stable angina at our institution from January 2015 to December 2020. Eligible patients started DAPT with aspirin and prasugrel and concomitantly received PPI therapy (lansoprazole or esomeprazole), with a follow-up period of two years. The primary endpoint was UGIB incidence, diagnosed during follow-up, serving as an efficacy measure. Secondary endpoints included the assessment of major bleeding (as defined by the Thrombolysis in Myocardial Infarction major bleeding criteria) and clinically relevant non-major bleeding events. Safety outcomes focused on adverse event incidence attributable to PPI use.

**Results:**

Among the 165 patients analyzed, 109 and 56 were included in the lansoprazole and esomeprazole groups, respectively, with cumulative incidence of UGIB at 96 weeks of 0.9% (1/109) and 3.6% (2/56). No significant differences in terms of major bleeding events or other bleeding outcomes were observed between the two groups. Adverse events related to PPI use were reported as diarrhea/soft stools in 7 (6%) cases and thrombocytopenia in 1 (1%) case in the lansoprazole group, whereas no such events were observed in the esomeprazole group. No clinically significant hematologic or biochemical abnormalities were reported.

**Conclusion:**

This study evaluated the efficacy and safety of PPIs in combination with DAPT, including prasugrel, following PCI, and suggests that lansoprazole and esomeprazole may offer comparable efficacy in preventing UGIB.

**Supplementary Information:**

The online version contains supplementary material available at 10.1186/s40780-024-00398-y.

## Introduction

Prompt percutaneous coronary intervention (PCI) has dramatically improved survival rates in patients with acute coronary syndromes (ACS), including myocardial infarction and unstable angina. Advances in drug-eluting stents (DES) have further decreased the risk of stent thrombosis, enabling shorter durations of dual antiplatelet therapy (DAPT) to minimize bleeding complications [1]. However, the co-administration of proton pump inhibitors (PPIs) is frequently necessary to mitigate the risk of antiplatelet agent-related upper gastrointestinal bleeding (UGIB) during DAPT.

Concerns have been raised about the potential increase in cardiovascular events when clopidogrel is combined with PPIs in the context of DAPT with aspirin and clopidogrel. However, the COGENT trial revealed that combining clopidogrel with a PPI significantly decreases the risk of gastrointestinal bleeding without adversely affecting major cardiovascular endpoints [2, 3]. Consequently, the American College of Cardiology/American Heart Association (2016) [4], European Society of Cardiology (2018) [5], and Japanese Circulation Society (2018) [6] guidelines recommend the use of PPI in patients at high risk for gastrointestinal bleeding on aspirin monotherapy or DAPT. Evidence supporting PPI use for primary prevention in patients with PCI without a history of peptic ulcers or UGIB remains insufficient despite these recommendations.

Clopidogrel’s antiplatelet efficacy depends on its conversion to an active metabolite by the CYP2C19 enzyme. The formation of the active metabolite is reduced in patients homozygous for non-functional CYP2C19 alleles, diminishing antiplatelet effects [7]. The likelihood of reduced clopidogrel efficacy is greater in Japanese patients, considering the higher prevalence of CYP2C19 poor metabolizers in the Japanese population compared to Western populations [8]. Conversely, prasugrel’s antiplatelet activity is not affected by genetic variations in CYP2B6, CYP2C9, CYP2C19, or CYP3A5, thereby maintaining consistent pharmacokinetic efficacy across different genotypes [9]. Additionally, concurrent PPI use does not affect prasugrel’s efficacy and bleeding risk [10]. Consequently, the combination of aspirin and prasugrel has become increasingly favored for DAPT after PCI in Japan.

However, prasugrel has been related to an increased risk of gastrointestinal bleeding (GIB) [11], and third-generation P2Y12 inhibitors have been generally associated with a higher GIB risk compared to clopidogrel [12]. It should also be taken into consideration that bleeding risks have been reported in post-marketing surveillance studies of prasugrel in large groups [13, 14]. This emphasizes the need for careful assessment of UGIB risk associated with PPI use in combination with aspirin/prasugrel-based DAPT.

This study conducted a retrospective analysis of cases at our institution where patients concomitantly received PPI with aspirin/prasugrel-based DAPT after PCI. This study aimed to evaluate the efficacy and safety of PPI use in preventing UGIB in this patient population.

## Methods

### Study population

This study included patients at Hokushin General Hospital who underwent PCI for ACS or stable angina, had a coronary stent placed, initiated DAPT with aspirin and prasugrel, and concomitantly started PPI therapy (either lansoprazole or esomeprazole) from January 1, 2015, to December 31, 2020, with a subsequent two-year follow-up. Only the first PCI was included among patients who underwent multiple PCIs within the study period. Lansoprazole and esomeprazole were the most predominantly used PPIs after PCI at our hospital; thus, cases using other PPIs, omeprazole and rabeprazole, or vonoprazan were excluded. Exclusion criteria were a history of active peptic ulcer disease use within 3 months of PCI, contraindications to aspirin or prasugrel (e.g., allergies), concurrent use of strong CYP3A4 inhibitors (ketoconazole, itraconazole, voriconazole, telithromycin, clarithromycin, ritonavir, saquinavir, nelfinavir, and atazanavir), inability to complete the two-year (96-week) follow-up after initiating DAPT, and discontinuation or change of PPI within two years without a clear reason. No restrictions were applied on DAPT duration in this study.

### Data collection

Medical records, including diagnoses, clinical laboratory values, prescription history, and medication counseling records by hospital pharmacists were used to investigate patient background and medication history. The patient background included age, sex, body mass index (BMI), Glasgow Blatchford Score (a risk score for acute upper gastrointestinal bleeding) [15], comorbidities (hypertension, diabetes mellitus, dyslipidemia, smoking, alcohol consumption, and chronic kidney disease, including hemodialysis status), gastrointestinal bleeding or ulcer history, concurrent medications (PPIs, H2-receptor antagonists, nonsteroidal anti-inflammatory drugs, anticoagulants, and antiplatelet agents other than aspirin and prasugrel), PPI type (including dosage), DAPT duration.

Efficacy and safety were evaluated based on diagnoses and clinical outcomes documented in the medical records. The primary efficacy endpoint was the incidence of UGIB diagnosed by physicians at 4, 12, 24, 48, 72, and 96 weeks. Secondary efficacy endpoints were major bleeding events (defined by Thrombolysis in Myocardial Infarction [TIMI] criteria as major bleeding) and clinically relevant non-major bleeding events. TIMI criteria defined major bleeding as “intracranial hemorrhage or clinically significant overt bleeding associated with a decrease in hemoglobin of > 5 g/dL or a decrease in hematocrit of > 15%.” Clinically relevant non-major bleeding was defined as “bleeding requiring medical or surgical intervention, unscheduled contact with a physician, treatment discontinuation or interruption, or bleeding causing pain or impairment in daily activities.”

The safety evaluation focused on PPI-related adverse events (interstitial pneumonia, thrombocytopenia, liver function abnormalities, anemia, diarrhea, pancytopenia, hyponatremia, etc.), comparing the incidence of these events during the 96-week observation period after PCI. Additionally, laboratory parameter changes, including hemoglobin, platelets, white blood cells (WBC), alanine transaminase (ALT), serum creatinine, serum sodium, serum potassium, serum magnesium, and serum calcium, were assessed before and at 96 weeks after therapy initiation.

### Statistical analysis

Continuous variables were presented as mean ± standard deviation or median (interquartile range) and compared using the Mann–Whitney U test or Wilcoxon signed-rank test. Categorical variables were presented as counts and percentages, and comparisons were conducted using Fisher’s exact test. A *p*-value of < 0.05 was considered statistically significant for all analyses. EZR software [16], which extends the functionalities of R and R Commander, was used for statistical analyses.

## Results

### Patient background

**Fig. 1:**
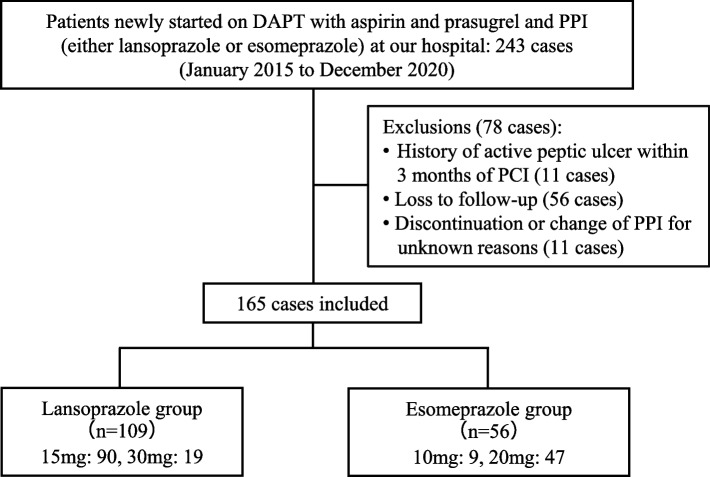
Selection flow in this study

Figure 1 illustrates the study flow and the number of patients receiving each PPI. Of the 243 patients who newly started DAPT with aspirin and prasugrel alongside PPI therapy, 78 were excluded, leaving 165 patients to be included in the study. Among them, 109 and 56 patients were included in the lansoprazole and esomeprazole groups, respectively. Table 1 presents the baseline characteristics of these patients. The median age was approximately 70 years, with approximately 70% being male and a mean BMI of approximately 24 kg/m2. A significant difference in terms of the prevalence of dyslipidemia was found between the lansoprazole and esomeprazole groups. However, other baseline characteristics were comparable between the groups. The duration of DAPT was shorter in the lansoprazole group.

**Table 1:** Characteristics of the patients at baseline

Characteristic	Lansoprazole group (*N* = 109)	Esomeprazole group (*N* = 56)	*P* value
Dosage-no. (%)	15 mg; 90 (82.6) 30 mg; 19 (17.4)	10 mg; 9 (16.1) 20 mg; 47 (83.9)	-
Median Age (yr)-(IQR)	71.0 (66.0–79.0)	68.0 (63.0–76.3)	0.209
Male-no. (%)	77 (71.0)	37 (66.1)	0.595
Median BMI (kg/m^2^)-(IQR)	24.3 (23.1–26.8)	24.9 (22.1–26.3)	0.539
Median GBS score- (IQR)	2 (0–3)	1 (0–2)	0.292
Acute coronary syndromes-no. (%)	73(67.0)	43(76.8)	0.212
Stable angina-no. (%)	36(33.0)	13(23.2)	0.212
Smoking history-no. (%)	23 (21.1)	15 (26.8)	0.441
Drinking history-no. (%)	47 (43.1)	25 (44.6)	1.000
History of present illness-no. (%)			
hypertension	70 (64.2)	38 (67.9)	0.730
diabetes	43 (39.4)	22 (39.3)	1.000
dyslipidemia	62 (56.9)	21 (37.5)	0.024
cronic kidney disese	8 (7.3)	4 (7.1)	1.000
hemodialysis	2 (1.8)	2 (3.6)	0.605
Past peptic ulcer	10 (9.2)	2 (3.6)	0.225
History of Medication-no. (%)			
H_2_ blocker	1 (0.9)	0 (0)	1.000
NSAIDs	4 (3.7)	3 (5.4)	0.690
anticoagulant	12 (11.0)	2 (3.6)	0.143
antiplatelet drug other than DAPT	5 (4.6)	6 (10.7)	0.186
Median duration of DAPT (day)-(IQR)	341 (205–426)	401 (333–535)	0.004
Median duration of SAPT (day)-(IQR)	314 (232–459)	280 (137–337)	0.019
SAPT using aspirin-no. (%)	93(85.3)	47(83.9)	0.822

### Efficacy outcomes

Table 2 presents the incidence of UGIB diagnosed by physicians at 4, 12, 24, 48, 72, and 96 weeks, which was the primary efficacy outcome. The cumulative incidence of UGIB at 96 weeks was 0.9% (1/109) and 3.6% (2/56) in the lansoprazole and esomeprazole groups, respectively.

**Table 2:** UGIB in follow-up

UGIB	Lansoprazole group (*N* = 109)	Esomeprazole group (*N* = 56)
after 4 week	0	0
after 12 week	1	0
after 24 week	0	1
after 48 week	0	1
after 72 week	0	0
after 96 week	0	0

Table 3 shows the secondary efficacy outcomes, including major bleeding events (as defined by TIMI criteria) and clinically relevant non-major bleeding events. No significant differences in terms of major bleeding events or other bleeding outcomes were found between the groups. Specific major bleeding events included one case in the lansoprazole of 15 mg group, where hemoglobin decreased after coronary artery bypass surgery, and two cases in the esomeprazole of 20 mg group, including one case of hemoglobin decrease due to melena and one case of subcutaneous hemorrhage after PCI. Other bleeding events were melena, hematochezia, and positive fecal occult blood test (8 cases), hematuria (3 cases), perioperative bleeding (4 cases), hemorrhoidal bleeding (1 case), and postmenopausal genital bleeding (1 case).

**Table 3:** Critical bleeding and other bleeding

	Lansoprazole group (*N* = 109)	Esomeprazole group (*N* = 56)	*P* value
Critical bleeding-no. (%)	1 (0.9)	2 (3.6)	0.266
Other bleeding-no. (%)	10 (9.1)	7 (12.5)	0.590

### Safety outcomes

Adverse events associated with PPI use were observed in 7 (6%) cases of diarrhea or soft stools and 1 (1%) case of thrombocytopenia in the lansoprazole group, with no such events reported in the esomeprazole group (Supplementary Table 1). The severity of diarrhea or soft stools in the lansoprazole group was categorized as grade 1–2 according to the Common Terminology Criteria for Adverse Events version 5.0. Table 4 presents clinical laboratory values before and at 96 weeks after PPI therapy initiation for patients in both the lansoprazole and esomeprazole groups. No clinically significant decreases in hemoglobin, thrombocytopenia, leukopenia, ALT elevation, creatinine increase, hyponatremia, hypomagnesemia, hypokalemia, or hypocalcemia were found at 96 weeks.

**Table 4:** Laboratory values before PPI initiation and at 96 weeks of treatment

Inspection item	Standard value	Group	0 week	*n*	96 week	*n*	*p*	
Hb (g/dL)	Male: 13.4–17.4 Female: 11.3–14.9	Lansoprazole	14.1 ± 0.17	108	13.9 ± 0.16	105	0.355	a
		Esomeprazole	14.1 ± 0.23	56	13.4 ± 0.27	56	0.001	b
PLT (× 10^4^/μL)	10.0–40.0	Lansoprazole	19.9 ± 0.53	108	18.9 ± 0.47	105	0.185	a
		Esomeprazole	19.8 ± 0.73	56	20.0 ± 0.73	56	0.326	b
WBC (/μL)	Male: 4100–8500 Female: 3900–7800	Lansoprazole	8223 ± 339	108	5864 ± 152	105	< 0.001	a
		Esomeprazole	9838 ± 972	56	6130 ± 241	56	< 0.001	b
ALT (U/L)	Male: 10–42 Female: 7–23	Lansoprazole	28.4 ± 1.8	102	21.7 ± 1.1	104	0.017	a
		Esomeprazole	27.6 ± 3.5	55	23.3 ± 1.9	53	0.832	a
Cr (mg/dL)	Male: 0.65–1.07 Female: 0.46–0.79	Lansoprazole	1.07 ± 0.11	109	1.08 ± 0.12	107	0.038	a
		Esomeprazole	1.24 ± 0.2	56	1.26 ± 0.19	56	0.051	b
Na (mEq/L)	138–145	Lansoprazole	140 ± 0.24	109	140 ± 0.24	107	0.023	a
		Esomeprazole	140 ± 0.35	55	141 ± 0.30	55	0.031	a
K (mEq/L)	3.6–4.8	Lansoprazole	4.1 ± 0.04	108	4.4 ± 0.04	106	< 0.001	a
		Esomeprazole	4.1 ± 0.07	55	4.3 ± 0.06	55	0.046	a
Mg (mg/dL)	1.7–2.5	Lansoprazole	2.1 ± 0.03	41	2.1 ± 0.03	51	0.502	a
		Esomeprazole	2.0 ± 0.08	14	2.0 ± 0.06	20	0.845	a
Ca (mg/dL)	8.8–10.1	Lansoprazole	9.3 ± 0.07	68	9.5 ± 0.05	74	0.092	a
		Esomeprazole	9.2 ± 0.07	29	9.3 ± 0.10	30	0.767	a

^a^Mann-Whitney U test

^b^Wilcoxon signed-rank sum test

## Discussion

Clinical trials that involved antiplatelet therapy after PCI revealed a lack of strategies to ensure appropriate gastric protection [17]. Consequently, efforts have been made to enhance guideline adherence by increasing the prescription rate of PPIs in patients on DAPT [18]. However, inappropriate PPI prescriptions pose a significant issue [19]. The present study analyzed lansoprazole and esomeprazole and revealed that the incidence of UGIB (Table 2) and bleeding events (Table 3) were similar between the two groups. The concomitant use of PPIs with P2Y12 inhibitors may provide gastrointestinal protection without adverse cardiovascular effects [20]. However, prasugrel administration in patients with ACS has caused a lower incidence of major adverse cardiovascular events (MACE) compared to clopidogrel and is also associated with a higher bleeding risk [21]. Therefore, the significance of PPI use in this study is considerable. Notably, the protective effect of PPI co-administration may be limited in patients receiving DAPT with a low risk of gastrointestinal bleeding, and reports suggest an increased risk of stroke and myocardial infarction under these circumstances [22]. These results underscore the need for further investigation.

Regarding safety, no clinically significant differences were found between lansoprazole and esomeprazole (Supplementary Table 1, Table 4). However, noteworthily, the lansoprazole group demonstrated a relatively higher, though mild, incidence of diarrhea or soft stools (6%) compared to the esomeprazole group. A significant decrease in WBC was observed, but it was considered a normal reduction after the elevation typically seen after acute myocardial infarction (AMI) (Table 4). The slight increases in creatinine and serum potassium levels may be related to the effect of renin-angiotensin inhibitors or aldosterone antagonists, which are initiated post-AMI, but this was not established in this study. Conversely, the indiscriminate use of PPIs carries risks, including reports of community-acquired pneumonia [23], Clostridium difficile infection [24], hypomagnesemia [25], and kidney impairment [26]. This study followed patients for two years after initiating DAPT, but further investigation is warranted to determine the optimal duration of PPI co-administration.

In Japan, several medications are approved for “the prevention of recurrent gastric or duodenal ulcers during low-dose aspirin (LDA) therapy,” including lansoprazole of 15 mg, rabeprazole of 5 mg (with a possible increase to 10 mg if the initial dose is insufficient), esomeprazole of 20 mg, and the potassium-competitive acid blocker, vonoprazan of 10 mg (Supplementary Table 2). Based on the study [27] showing that the incidence of gastrointestinal bleeding was 8.0% (16/199) in the PPI non-use group (199 patients) and 3.9% (4/103) in the rabeprazole group (103 patients) for DAPT with aspirin and clopidogrel, this study revealed no significant differences in efficacy or safety between lansoprazole and esomeprazole. As of August 2024, the cost per tablet for esomeprazole of 20 mg is 41.8 JPY, compared to 12.4 JPY for lansoprazole of 15 mg. Although our study’s sample size limits the ability to conclude equivalency in efficacy and safety, our findings underscore the potential value of future research on the cost-effectiveness of PPIs in this setting.

Finally, the limitations of this study include its retrospective design, single-center setting, and the limited number of cases. With the trend towards shorter durations of DAPT administration [1], the significance of PPI administration in patients with a low risk of gastrointestinal bleeding requires further investigation. In addition, it is necessary to consider stratified analyses based on age and the impact of concomitant medications. However, this study is significant as the first to assess the efficacy and safety of PPIs for primary prevention during DAPT, including prasugrel. We hope that this study will contribute to further research in this field.

## Conclusion

This study evaluated the efficacy and safety of PPIs, specifically lansoprazole and esomeprazole, when used in combination with DAPT, including prasugrel, after PCI. Our findings suggest similar efficacy and safety between the two PPIs in preventing UGIB. Future research, particularly multi-center collaborative studies, should focus on the cost-effectiveness of PPIs to guide optimal therapeutic strategies.

## Supplementary Information

Supplementary Material 1.

## Data Availability

Data will be made available on request.

## Abbreviations

ACS: Acute coronary syndromes
AMI: Acute myocardial infarction
BMS: Bare metal stent
DAPT: Dual antiplatelet therapy
DES: Drug-eluting stent
GIB: Gastrointestinal bleeding
LDA: Low-dose aspirin
MACE: Major adverse cardiovascular events
P-CAB: Potassium-competitive acid blocker
PCI: Percutaneous coronary intervention
PPI: Proton pump inhibitor
TIMI: Thrombolysis in myocardial infarction
UGIB: Upper gastrointestinal bleeding
