# Supplementary material for: The risk of upper gastrointestinal bleeding associated with concomitant proton pump inhibitor administration during dual antiplatelet therapy with aspirin and prasugrel: a retrospective single-center study
Source: J Pharm Health Care Sci. 2024 Nov 25;10:76. doi: 10.1186/s40780-024-00398-y (PMC11587642; doi:10.1186/s40780-024-00398-y)
Supplement: Supplementary file 1 — Supplementary Material 1. [file 40780_2024_398_MOESM1_ESM.pdf]

Supplementary data

The risk of upper gastrointestinal bleeding associated with concomitant proton pump inhibitor administration during dual antiplatelet therapy with aspirin and prasugrel: A retrospective single-center study

Yutaro Ide[1], Go Morikawa[1*], Kyohei Yoshida[1], Yuki Takano[1], Ken Kubota[1], Katsuko Okazawa[1] and Takeo Yasu[2]

[1] *Department of Pharmacy, Hokushin General Hospital, Nagano, Japan.*
[2] *Department of Medicinal Therapy Research, Pharmaceutical Education and Research Center, Meiji Pharmaceutical University, Tokyo, Japan.*

[*] Corresponding author. E-mail address: gomorikawa@hokushin-hosp.jp (G. Morikawa). Department of Pharmacy, Hokushin General Hospital, 1-5-63, Nishi, Nakano, Nagano 383-8505, Japan.
Present affiliation is Division of Legal Medicine, Department of Forensic Science, Iwate Medical University, 1-1-1 Idaidori, Yahaba-cho, Shiwa-gun, Iwate 028-3694, Japan.
E-mail address: gomorikw@iwate-med.ac.jp

Supplementary Table 1 Major adverse events

	Lansoprazole group (N=109)	Esomeprazole group (N=56)	*P* value
Diarrhea and soft stool－no. (%)	7　(6%)	0　(0%)	0.097
Thrombocytopenia－no. (%)	1　(1%)	0　(0%)	1.000
Total－no. (%)	8　(7%)	0　(0%)	0.052

Supplementary Table 2 List of Main Proton Pump Inhibitors (PPIs) Available in Japan

Brand name		Lansoprazole OD Tablets	Rabeprazole Na Tablets	Esomeprazole Capsules	Takecab OD Tablets
Active ingredient		Lansoprazole	Rabeprazole sodium	Esomeprazole magnesium hydrate	Vonoprazan fumarate
Dosage：Drug prices※		15 mg: 12.4 JPY<br>30 mg: 20.8 JPY	5 mg: 6.9 JPY<br>10 mg: 13.8 JPY<br>20 mg: 20.7 JPY	10 mg: 24.0 JPY<br>20 mg: 41.8 JPY	10 mg: 96.8 JPY<br>20 mg: 144.8 JPY
Indications	1. Gastric ulcer	○	○	○	○
	2. Duodenal ulcer	○	○	○	○
	3. Anastomotic ulcer	○	○	○	
	4. Reflux esophagitis	○	○	○	○
	5. In maintenance therapy for recurrent or relapse reflux esophagitis	○	○ (10 mg)	○	○
	6. Zollinger-Ellison syndrome	○	○	○	
	7. Non-erosive gastroesophageal reflux disease	○ (15 mg)	○ (10 mg)	○ (10 mg)	
	8. Eradication of Helicobacter	○	○ (10 mg)	○	○ (20 mg)
	9. Suppression of recurrence of gastric/duodenal ulcer associated with low-dose aspirin	○ (15 mg)	○<br>(5 mg, 10 mg)	○ (Adults: 20 mg; children: 10 mg)	○ (10 mg)
	10. Suppression of recurrence of gastric/duodenal ulcer associated with nonsteroidal anti-inflammatory drug	○ (15 mg)		○ (Adults: 20 mg; children: 10 mg)	○ (10 mg)

※ "The drug prices are the official prices per tablet (or capsule). The prices listed are as of August 2024 in Japan. If multiple brands with the same active ingredient are available, the price of the least expensive option is listed."
